# Supplementary figures and images for: Quantifying the Interplay between Environmental and Social Effects on Aggregated-Fish Dynamics
Source: PLoS One. 2011 Dec 12;6(12):e28109. doi: 10.1371/journal.pone.0028109 (PMC3236193; doi:10.1371/journal.pone.0028109)

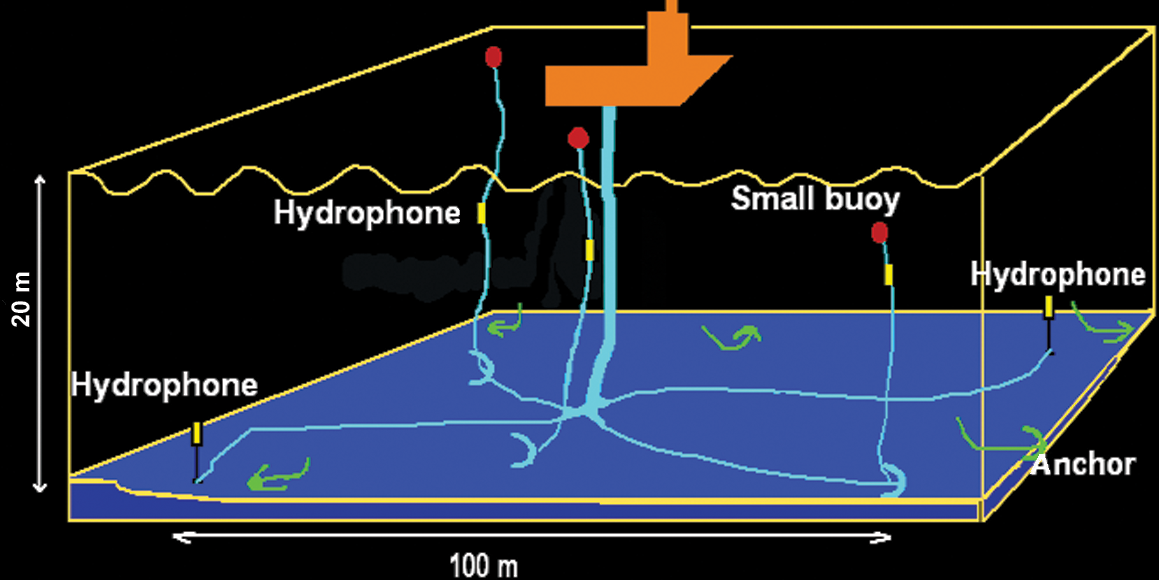

Supplement: Figure S1 — The HTI™ experimental setting. The boat, with the cables underneath, represents the floating object or ‘FAD’. (TIF) [file pone.0028109.s001.tif]

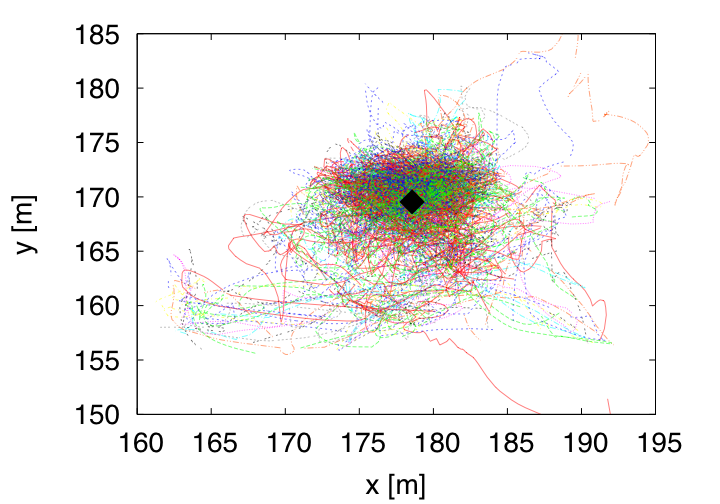

Supplement: Figure S2 — Trajectories of the tracked fish in the plane around the FAD from 13:00 to 14:00 Different colors indicate different fish and black point indicates the FAD position. (TIF) [file pone.0028109.s002.tif]

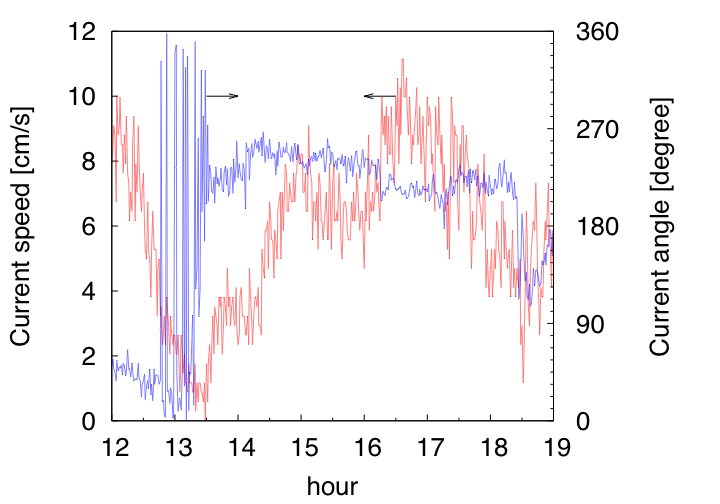

Supplement: Figure S3 — Current speed (red line) in cm/s and current angle (blue line), with respect to the North (East = 90; West = 270) recorded during the experiment. (TIF) [file pone.0028109.s003.tif]

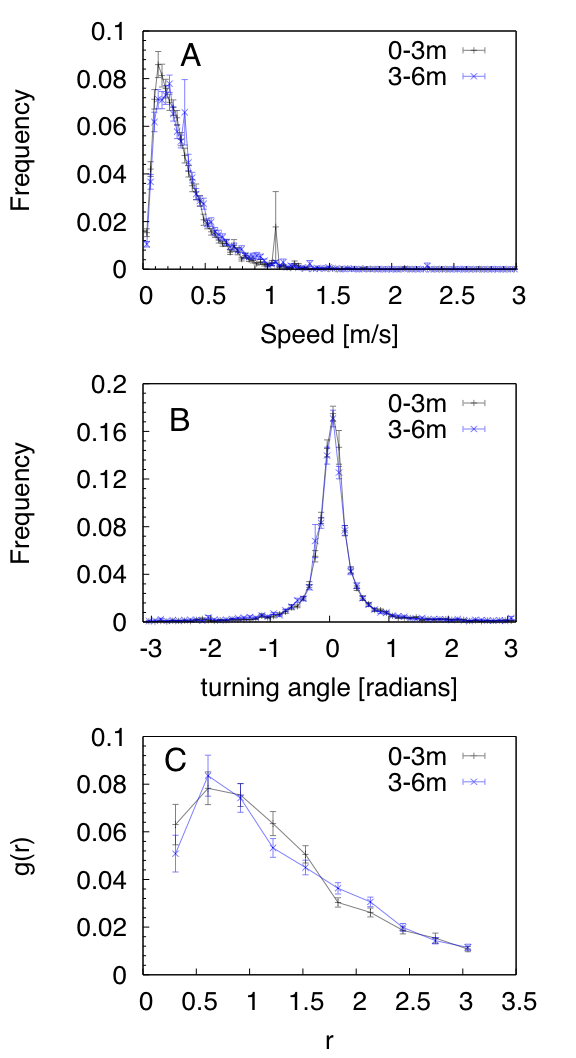

Supplement: Figure S4 — Swimming speed distribution (A), turning angle distribution (B) and pair-correlation function (C), calculated at different radial distances from the FAD. (TIF) [file pone.0028109.s004.tif]
